# Should ApoE4 gene editing therapy be approached with caution when AD is accompanied by retinal neurodegenerative diseases?

**DOI:** 10.3389/fneur.2026.1934307

**Published:** 2026-10-01

**Authors:** Yi Wenhua, Ma Wenjing, Dai Yan, Li Fei, Chen Juan, Tang Xia, Guan Yu, Li Xin, Huang Yanlin, Guo Ya, Yu Xuelin, Luo Jing, Jiang Meimin, Li Wenyi, Kang Yilin, Liu Hongke, Li Yating

**Affiliations:** 1 Chengdu Fifth People's Hospital, Chengdu, China; 2 Hospital of Chengdu University of Traditional Chinese Medicine, Chengdu, China

**Keywords:** AD, AMD, ApoE4, DR, gene editing, glaucoma, ocular safety

## Abstract

Apolipoprotein E4 (ApoE4) is the strongest genetic risk factor for Alzheimer’s disease (AD), and ApoE4-directed gene editing and gene therapy have emerged as promising therapeutic strategies for AD, although they remain investigational and are not yet an established mainstream treatment. Interestingly, however, ApoE4 is reported to be associated with a reduced risk of retinal degenerative diseases such as glaucoma, age-related macular degeneration (AMD), and diabetic retinopathy (DR). Therefore, should ApoE4 gene editing therapy be approached with caution when AD occurs concurrently with glaucoma, AMD, or DR? In view of these and based on the existing literature, the present study detailed the distinct roles of ApoE4 in AD, glaucoma, AMD, and DR, with a purpose to offer valuable insights into subsequent ApoE4 gene editing therapies. We also summarise the ApoE-targeted therapeutic programmes currently in preclinical and early clinical development, delineate the remaining translational challenges, and discuss how patients with coexisting retinal neurodegenerative disease might be evaluated and monitored.

## 1 Introduction

Alzheimer’s disease (AD) is the most common cause of dementia (1), whereas glaucoma, age-related macular degeneration (AMD) and diabetic retinopathy (DR) are leading causes of irreversible vision loss (2). Although these disorders affect different organs, all are characterised by progressive neurodegeneration, and increasing evidence indicates that they share molecular mechanisms, including lipid dysregulation, impaired proteostasis and chronic inflammation (3). Apolipoprotein E (ApoE), the principal lipid carrier of the central nervous system (CNS) and of the retina, is one of the few molecules implicated in all of them. This review therefore asks a clinically relevant question: when AD and a retinal neurodegenerative disease coexist, can an ApoE-directed intervention be expected to benefit both? (4).

Apolipoprotein E (ApoE) is a 34 kDa polymorphic protein comprising 299 amino acids (5–8). It primarily consists of three domains, including the N-terminal domain (residues 1–167), a four-helix bundle responsible for receptor binding; the C-terminal domain (residues 206–299), a three-helix bundle responsible for lipid binding; and a flexible hinge region (residues 167–206) connecting them (9–12). As a crucial component of lipoprotein particles (LPPs), ApoE can facilitate lipid transfer—that is, the movement of cholesterol and other lipids between cells within lipoprotein particles—by serving as a recognition ligand for specific cell surface receptors (13). Following the acquisition of cholesterol from cells with excess cholesterol, these lipid particles can bind and internalize ApoE-containing LPPs. When it was reported for the first time by Virgie G. Shore and Bernard Shore in 1973 (14), ApoE was initially discovered to interact with the low-density lipoprotein (LDL) receptor and was identified as a key regulator of lipoprotein metabolism, exhibiting an intimate association with the pathogenesis of type III hyperlipoproteinemia (15). With extensive and in-depth research, ApoE has been discovered to be involved in axonal and myelin regeneration after central nervous tissue injury, exerting mediating roles in neuronal repair, remodeling, and protection in neurodegenerative diseases (6, 16).

From the aspect of anatomy, peripheral ApoE cannot enter the central nervous system due to the presence of the blood–brain barrier (BBB) and blood-retinal barrier (17). In the brain, ApoE is primarily produced by glial cells—above all astrocytes, and by microglia under stress or injury—but not by neurons under physiological conditions; neurons can, however, upregulate ApoE expression under stress or injury, while in the retina, ApoE is mainly produced by Müller cells (18–20). The two compartments also differ in their cellular sources and developmental dynamics. During mouse retinal development, lipoprotein production shifts from ApoA-I to ApoE between postnatal days 5–7 (20), during which the production of ApoE becomes highly concentrated in Müller cells (21, 22). At this stage, Müller cells interact with adjacent LRP1-expressing plexiform layers and photoreceptor cells, which are responsible for escorting LRP1-expressing cells to facilitate synapse formation by supplying ApoE and cholesterol (20, 23–25). Additionally, astrocytes, microglia, and neurons themselves can also produce ApoE, and the resultant ApoE further bind to cholesterol to form ApoE-cholesterol complexes, which are internalized into retinal ganglion cells (RGCs) through LDL receptors (18, 26). ApoE acts by binding to a family of cell-surface receptors: the primary membrane receptors for ApoE belong to the LDL receptor (LDLR) family-related proteins (LRPs) (26), which comprises the canonical LDLR together with several LDLR-related proteins such as LDLR-related protein 1 (LRP1), megalin, very-low-density lipoprotein receptor (VLDLR), ApoE receptor 2 (apoER2), and LRP5/6 are also members of this family, in addition to the canonical LDLR (27–32). ApoE and its LRPs are closely associated with cellular differentiation, neuroprotection, and axonal growth in RGCs (33–36).

ApoE is produced by three alleles-ε4, ε3, and ε2-at a single gene locus. Three homozygous types (E4/4, E3/3, and E2/2) and three heterozygous types (E4/3, E4/2, and E3/2) yielded owing to the combination of these alleles. The only difference of the three alleles, ApoE4 (Arg-112, Arg-158), ApoE3 (Cys-112, Arg-158), and ApoE2 (Cys-112, Cys-158), lies in the single amino acid substitutions at residues 112 and 158 (36). Structural differences arising from various genotypes may trigger distinct lipid-binding capacities, abilities to incorporate into LPPs, and receptor affinities. ApoE4 adopts a pathological conformation in its N-terminal and C-terminal domains, respectively, in the context of interactions between arginine (Arg) and glutamic acid (37). This inter-domain interaction can further render ApoE4 to proteolytic cleavage, and the resulting fragments may be important in ApoE4 neurotoxicity (38).

The ApoE4 allele is the strongest genetic risk factor for Alzheimer’s disease (AD). At least one E4 allele may be carried by approximately 65–80% of AD patients (39–44), and the degree of risk exhibits a gene dose-dependent effect, with up to 15-fold increased risk of developing AD in the presence of E4/E4 homozygotes (45–47). Therefore ApoE-directed interventions—including gene editing, gene replacement and RNA-based suppression of ApoE4—are being actively explored. These are emerging and promising strategies, but they remain at a preclinical or early clinical stage and do not yet constitute a mainstream therapeutic strategy for AD (47–51). However, it is interesting to note that ApoE4 is associated with a reduced risk of retinal degenerative diseases such as glaucoma, age-related macular degeneration (AMD), and diabetic retinopathy (DR) (19, 52–57). Why the same allele is detrimental in AD, but protective in the aforementioned retinal degenerative diseases, which is pending further systematic investigation. Moreover, for AD patients suffering from glaucoma, AMD, or DR concurrently, while ApoE4-targeted therapy may be beneficial for AD, it should be clarified whether this treatment affects the progression of these three conditions. In view of these and based on the existing literature, the present study detailed the differential roles of ApoE4 in AD, glaucoma, AMD, and DR, with the goal of offering valuable insights into future ApoE4 gene-editing therapies.

## 2 The role of ApoE4 in AD

AD is primarily characterized by two pathological hallmarks: senile plaques (SP) and neurofibrillary tangles (58, 59). These two features lead to the formation of two central hypotheses in the pathogenesis of AD, namely, the amyloid-beta (Aβ) cascade and Tau hyperphosphorylation. In the progression of AD, ApoE4 has been discovered to mediate multiple pathological processes, such as Aβ pathology, Tau phosphorylation, synaptic damage, oxidative stress, mitochondrial dysfunction, and neuroinflammation, each is discussed in turn below, and readers seeking a more comprehensive treatment of individual topics are referred to dedicated reviews.

### 2.1 ApoE4 promotes Aβ production and toxicity

A substantial body of evidence supports a single conclusion: ApoE4 increases both the amount of Aβ produced and the toxicity of the species that are formed (60). Specifically, ApoE4 promotes Aβ production through several independent mechanisms and favours the assembly of oligomeric and protofibrillar conformers, which are generally considered the most synaptotoxic forms (61). ApoE4 may escalate the risk of AD by initiating and accelerating the production, aggregation, and deposition of Aβ within the brain (62). Aβ is produced through the hydrolysis of amyloid precursor protein (APP) by β- and γ-secretases, and can polymerize into amyloid fibrils, main components of SP in AD pathology.

On one hand, ApoE4 can enhance LDLR-related protein 1 (LRP1)-mediated endocytic trafficking of APP to enhance the production of Aβ (63, 64). On the other hand, compared to ApoE2 and ApoE3, ApoE4, as a signaling molecule for Aβ, can bind to ApoE receptors to facilitate the interaction with dual leucine zipper kinase more readily, thereby activating ERK1/2 MAP kinases and inducing c-Fos phosphorylation (65). It may subsequently stimulate the transcription factor AP-1, increase APP transcription, and eventually boost Aβ production. With elevated intracellular APP, ApoE4 and 27-hydroxycholesterol can synergistically activate the C/EBPβ/δ-secretase signaling pathway in neurons (66). Activated δ-secretase can further leak into the cytoplasm, cleaving multiple substrates including APP and Tau, promoting Aβ production as well as Tau proteolytic cleavage and aggregation, ultimately driving AD pathogenesis (66). Moreover, ApoE4 can promote the formation of Aβ oligomers in the brain more significantly. Compared to ApoE2 and ApoE3, as evidenced by the results of flow cytometry in primary immortalized astrocytes, there was a significant interaction between ApoE4 and Aβ, making this interaction more likely to form profibrillar deposit complexes (67, 68). Taken together, ApoE4 acts at several consecutive steps—APP trafficking and transcription, secretase-mediated processing, and extracellular assembly—so that both the quantity and the aggregation propensity of Aβ are increased.

### 2.2 ApoE4 promotes tau hyperphosphorylation

Overall, ApoE4 is associated with a heavier burden of Tau pathology in AD, and this association is gene-dose dependent. Compared to patients without the ApoE4 allele, ApoE4 allele-harbored AD patients may experience worsened pathophysiology of neurofibrillary tangles (NFTs) in the cerebral cortex (69). Regionally, this burden is not uniform: AD patients with the expression of ApoE4 exhibit a relatively higher density of NFTs in the medial temporal lobe, particularly in the entorhinal cortex (70). At the molecular level, an astrocyte-derived cofactor contributes to this effect: GPC-4 from the glypican family is an astrocyte-secreted protein that can induce the accumulation and propagation of Tau (71). GPC-4 exhibits a stronger binding affinity for ApoE4, and preferentially interacts with ApoE4 to promote Tau hyperphosphorylation (72). Hyperphosphorylated Tau is the primary component of NFTs, and the clearance of extracellular Tau is itself receptor-dependent: hyperphosphorylated Tau can be internalised and degraded through LRP1-mediated endocytosis. Because ApoE4 has a higher affinity for LRP1 than hyperphosphorylated Tau, it outcompetes Tau for this clearance route, resulting in inefficient binding of hyperphosphorylated Tau to LRP1 and impaired Tau degradation (73). Thus, ApoE4 amplifies Tau pathology both by promoting its phosphorylation and by impeding its receptor-mediated clearance.

### 2.3 ApoE4 promotes synaptic damage

Synaptic loss is the pathological correlate that most closely tracks cognitive decline, and ApoE4 compromises synapses through both receptor-trafficking and vascular routes. ApoE4 may impair lipoprotein transport on neuronal surfaces to induce the shortening of dendrite length and the reduction of dendritic spine density, thereby promoting early cognitive impairment in AD and synaptic damage (74, 75). Reelin is a neuroprotein with crucial role during neurodevelopment, which can serve as a regulator of brain development and synaptic strength (74). Its ability to enhance synaptic glutamate receptor activity can be significantly diminished by ApoE4 (74). A second, vascular route operates in parallel. It can be interpreted that ApoE4 can reduce the neuronal surface expression of Apoer2 (a dual-function receptor for both ApoE and Reelin) as well as NMDA and AMPA receptors by sequestering them in intracellular compartments, thereby triggering synaptic damage (74). Additionally, ApoE4 can activate the cyclophilin A-matrix metalloproteinase 9 (CypA-MMP9) pathway to accelerate the disruption of the BBB and promote neuronal synaptic dysfunction (76). In short, ApoE4 deprives the synapse of trophic receptor support while simultaneously exposing it to a compromised barrier environment.

### 2.4 ApoE4 promotes oxidative stress

The brain in AD patients exists in a state of high oxidative stress, and ApoE4 allele carriers are associated with elevated levels of oxidative stress, which is thought to be a possible contributor to the earlier onset of AD (77). The degree of oxidative stress in the AD-attacked brain exhibits an intimate association with the ApoE genotype in the following order of ApoE2 < ApoE3 < ApoE4 (77). In synaptosomes isolated from ApoE4 mice (i.e., mice expressing human ApoE4, relative to ApoE3 controls), there were consistently increased levels of reactive oxygen species (ROS) and the oxidation of proteins and lipids (78). Compared to AD patients without the ApoE4 allele, ApoE4 allele carriers were detected with higher levels of hydroxyl radicals in the blood and lower cerebral oxygen utilization rates (79). The state of high oxidative stress can promote the upregulation of the regulator of calcineurin 1 (RCAN1) (80). Specifically, RCAN1 upregulation can suppress calcineurin to weaken the dephosphorylation of Tau (80). Simultaneously, RCAN1 activation can increase the level of glycogen synthase kinase 3 (GSK3) (80). Elevated GSK3 will further promote Tau phosphorylation, thereby facilitating the formation of NFTs in AD patients (81). Viewed as a whole, these findings suggest a coherent cascade: oxidative stress induced by ApoE4 upregulates RCAN1, which simultaneously suppresses calcineurin-mediated dephosphorylation of Tau and increases GSK3, thereby providing a mechanistic bridge between oxidative stress and Tau hyperphosphorylation.

### 2.5 ApoE4 contributes to mitochondrial dysfunction

Mitochondrial failure is not merely a consequence but an early driver of AD pathogenesis, and ApoE4 targets mitochondrial quality control at several levels. Mitochondrial dysfunction has been recognized to be a driver of the early stage of AD pathogenesis. ApoE4 can modulate mitochondrial-related functions to promote AD progression (82). ApoE4 significantly impairs neuronal mitochondrial function both *in vivo* and *in vitro* by triggering endoplasmic reticulum (ER) stress, upregulating GRP75, and subsequently increasing mitochondria-associated ER membrane formation. Furthermore, ApoE4 can inhibit FoxO3a in the brain and interfere with key mitophagy pathways such as PINK1/Parkin and BNIP3 to disrupt mitophagy (83–85). Besides, ApoE4 may induce mitochondrial dysfunction by impairing the activities of mitochondrial respiratory complexes III and IV (86). Collectively, ApoE4 both damages mitochondria and prevents their removal, so that dysfunctional organelles accumulate.

### 2.6 ApoE4 exacerbates neuroinflammation

ApoE4 can also act as an intensifying factor of neuroinflammation through the exacerbation of plaque-induced microglial reactivity and disruption of lipid metabolism, i.e., it amplifies the inflammatory response of microglia to existing pathology while simultaneously disturbing glial lipid handling. Compared to mice carrying ApoE2 and ApoE3, microglia in mice harboring ApoE4 were detected with an abnormal pro-inflammatory secretory profile, manifesting as more pronounced and sustained increase in the production of inflammatory factors (NO, IL-1β, IL-6, and TNF-α), while significantly reduced anti-inflammatory cytokines such as IL-4. It may consequently enhance the susceptibility to inflammation and is positively correlated with gene dosage (87–90). Furthermore, ApoE4 can induce the activation of Ca^2+^-dependent phospholipase A2 (cPLA2). There may be enhanced translocation of cPLA2, upon activation, to the cytoplasm, which may further boost the release of arachidonic acid and exacerbate neuroinflammation ultimately (91, 92).

Triggering receptor expressed on myeloid cells 2 (TREM2), expressed on microglia, is an immune receptor, which has also be found to be a major genetic risk factor for AD development (92). It has an association with microglial inflammatory responses, including proliferation, migration, and phagocytosis (93). ApoE4 demonstrates the highest affinity for TREM2 compared to ApoE2 and ApoE3, which may underlie the ApoE4-associated risk in AD (94). The increased affinity of TREM2 for ApoE4 may amplify TREM2 signaling and lead to microglial overactivation, thereby elevating AD risk. The ApoE-TREM2 pathway can be activated in the presence of neuronal cell death, inducing an imbalance in microglial homeostasis and driving their transformation into the disease-associated microglial neurodegenerative phenotype (MGnD), thereby exacerbating neuroinflammation (95). Accordingly, ApoE4 does not initiate neuroinflammation *de novo* but lowers the threshold for, and amplifies the duration of, microglial activation.

### 2.7 Other effects of ApoE4 in AD

ApoE4 plays significant roles in multiple aspects of AD pathogenesis, such as sleep disturbances and impaired cerebrovascular integrity in AD patients.

Regarding sleep disturbances, which are both an early manifestation and a potential modifier of AD risk: ApoE4 allele-harboring older adults with cognitive impairment typically exhibit longer sleep duration (96). Homozygous carriers for ApoE4 allele demonstrate more pronounced sleep abnormalities (97). Improving sleep quality may mitigate the negative impact of ApoE4 on NFTs (98). Additionally, sleep disturbances may activate microglia and even induce neuroinflammation (98). Sleep disturbance is therefore best regarded as a potentially modifiable amplifier of ApoE4-related pathology rather than a mere epiphenomenon.

A second “other” domain concerns the cerebral vasculature, which is closely intertwined with the amyloid and Tau pathways described above. In terms of cerebrovascular integrity, ApoE4 may exacerbate the deposition of Aβ in the brain, triggering the occurrence of cerebral amyloid angiopathy (CAA), resulting in serious compromise of the vascular integrity and BBB function (99, 100). Compared to ApoE3, ApoE4 may accelerate the breakdown of BBB, neuronal loss, and behavioral deficits, independent of Aβ. ApoE4 can also activate the CypA-MMP9 pathway in the cerebrospinal fluid to accelerate BBB metabolic breakdown, which may contribute to neuronal and synaptic dysfunction (101).

In addition to these mentioned above, cerebral glucose metabolism, TAR DNA binding protein-43 pathology, and its relationship with other non-AD diseases are poorly understood at present and deserve further investigation in the future (102–106).

Since the topic of APOE in AD is broad, this subsection is selective rather than exhaustive: here, we highlight representative primary studies; for those who wish to learn more, detailed reviews on this aspect can be found in the following three articles: *ApoE in Alzheimer’s Disease: Pathophysiology and Therapeutic Strategies, published in Molecular Neurodegeneration, 2022; Apolipoprotein E Isoform-Dependent Effects on the Processing of Alzheimer’s Amyloid-β, published in Biochimica et Biophysica Acta (BBA) – Molecular and Cell Biology of Lipids, 2021;* and *Roles of ApoE4 on the Pathogenesis in Alzheimer’s Disease and the Potential Therapeutic Approaches, published in Cellular and Molecular Neurobiology, 2023* (see Figure 1).

**Figure 1:**
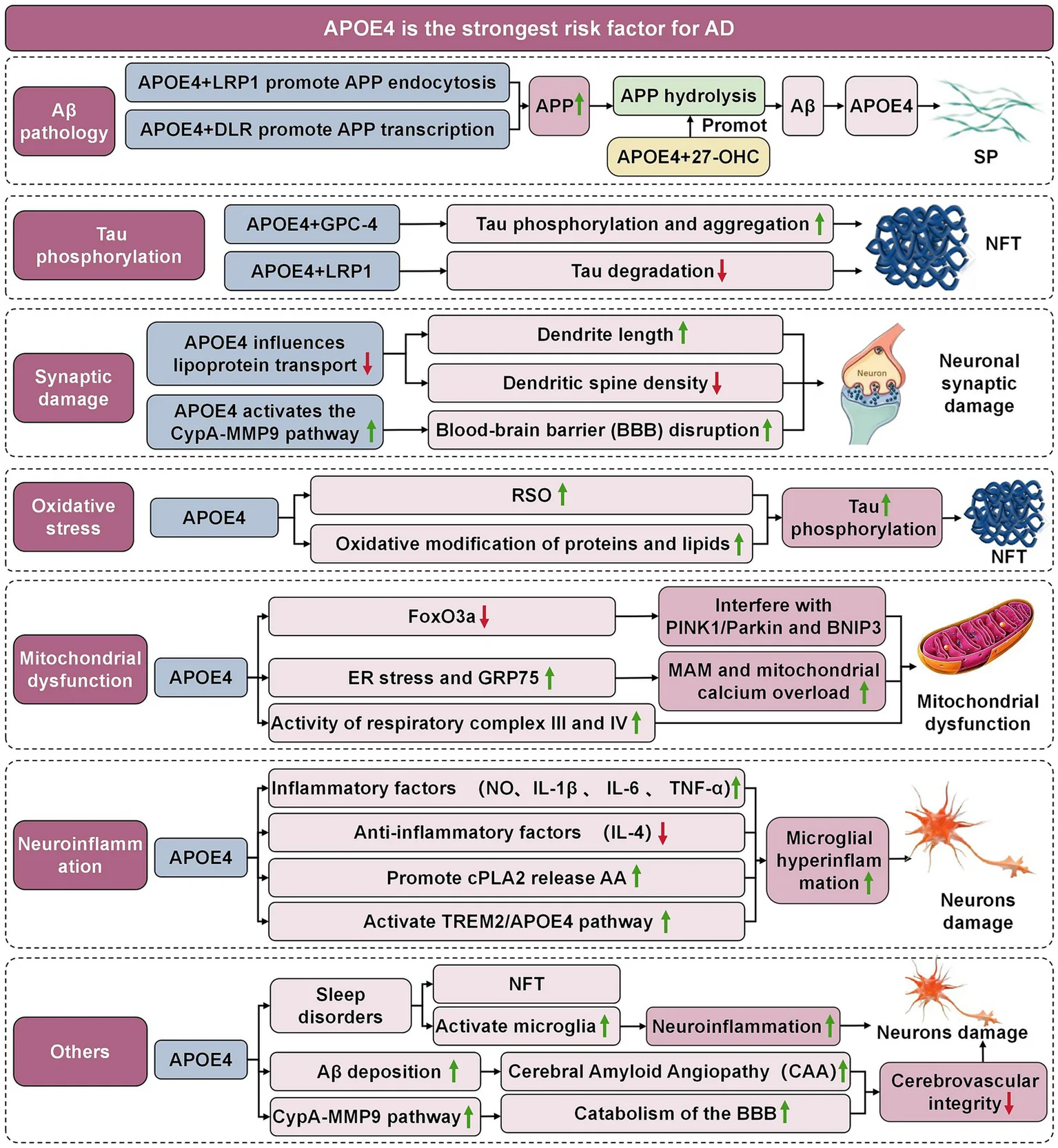
Schema 1: schematic overview of the ApoE4-driven pathological cascades in AD. ApoE4 promotes Aβ pathology (LRP1-mediated APP endocytic trafficking, APP transcription via ERK1/2–AP-1, δ-secretase activation and assembly of oligomeric/protofibrillar species); Tau hyperphosphorylation and NFT formation; synaptic damage (reduced neuronal surface ApoER2, NMDA and AMPA receptor expression, shortened dendrites and reduced spine density); blood–brain barrier disruption via the CypA–MMP9 pathway; oxidative stress; mitochondrial dysfunction; and neuroinflammation. Arrowheads indicate promotion and blunt ends indicate inhibition (this schema is referred to in Section 1).

## 3 The role of the ApoE4 allele in retinal degenerative diseases

The sections above describe ApoE4 as a risk allele in the brain. In the retina, by contrast, the same allele has repeatedly been associated with neutral or even favourable outcomes, a paradox that is central to the question posed by this review.

### 3.1 Role of the ApoE4 allele in glaucoma

Glaucoma is a retinal neurodegenerative disease characterized by the progressive loss of RGCs. At present, there are still conflicting results across clinical cohorts regarding the association between ApoE4 and glaucoma. For instance, Mullany S et al. reported no association between ApoE4 and glaucoma in a multi-cohort study of European ancestry populations (107). In contrast, a number of other cohorts suggest that ApoE4 may increase risk or accelerate progression in specific subgroups, for example in women or in normal-tension glaucoma, while still others report a protective effect; these divergent findings are considered in turn below.

On the one hand, several studies support a detrimental role of ApoE4 in defined subpopulations. Furthermore, ApoE4 might accelerate the progression of glaucoma in women during perimenopause, as suggested by a cross-sectional study in a UK population by Shi et al. (108). A retrospective analysis of a prospective Australian cohort by Marshall H et al. demonstrated that ApoE4 could accelerate the rate of retinal thinning, particularly in normal tension glaucoma (NTG) (109). Vickers et al. revealed in an association study in a Tasmanian population that ApoE4 was associated with an increased risk of NTG (110). Furthermore, in another association study in a population from Northeast China by Yuan et al., ApoE4 was discovered to be a potential risk factor for primary glaucoma, while the ApoE2 allele might be a protector in primary open-angle glaucoma (POAG) (111).

On the other hand, the largest available datasets support a protective role. Liuska PJ et al. conducted a large-scale association analysis of 412,171 cases in a Finnish population, with the confirmation of a negative correlation of ApoE4 with overall glaucoma, NTG, and POAG, suggesting a protective role of ApoE4 against glaucoma (112). An association study in Massachusetts by Margeta MA et al. also demonstrated that ApoE4 can reduce the risk of POAG (19). Similarly, a cross-sectional study on a Chinese population by Lam CY et al. indicated that ApoE4 can lower the risk of NTG, yet without obvious association with POAG (113).

Noticeably, these clinical cohort studies were performed based on populations from different countries, even without the classification of glaucoma subtypes in some studies. It necessitates further exploration to unveil whether the presence of such contradictory results is related to regional populations and glaucoma subtypes.

Beyond these, a recent animal study also yielded compelling evidence supporting the protective role of ApoE4 in glaucoma. Galectin-3, the only chimeric member of the β-galactoside-binding lectin family, functions significantly in proliferation, apoptosis, angiogenesis, phagocytosis, inflammation, and immune responses (114–118). By binding to microglial receptors TREM2 and Toll-like receptor 4 (TLR4), it can sustain paracrine activation of TLR4 in surrounding microglia and promote inflammatory responses in microglia across various neuroinflammatory diseases (114–116, 119–125). In retinal diseases, it can directly promote the apoptosis of RGCs (126, 127). In addition, Margeta MA et al. demonstrate in an animal experiment that ApoE4 can inhibit the activation of microglia into the disease-associated MGnD, reduce Galectin-3 expression, and promote RGC survival (127). Thus, in glaucoma the available evidence spans the full spectrum from harmful to protective, and the direction of the association appears to depend on the population studied, the glaucoma subtype and, in the experimental setting, on the inflammatory phenotype of retinal microglia (see Figure 2).

**Figure 2:**
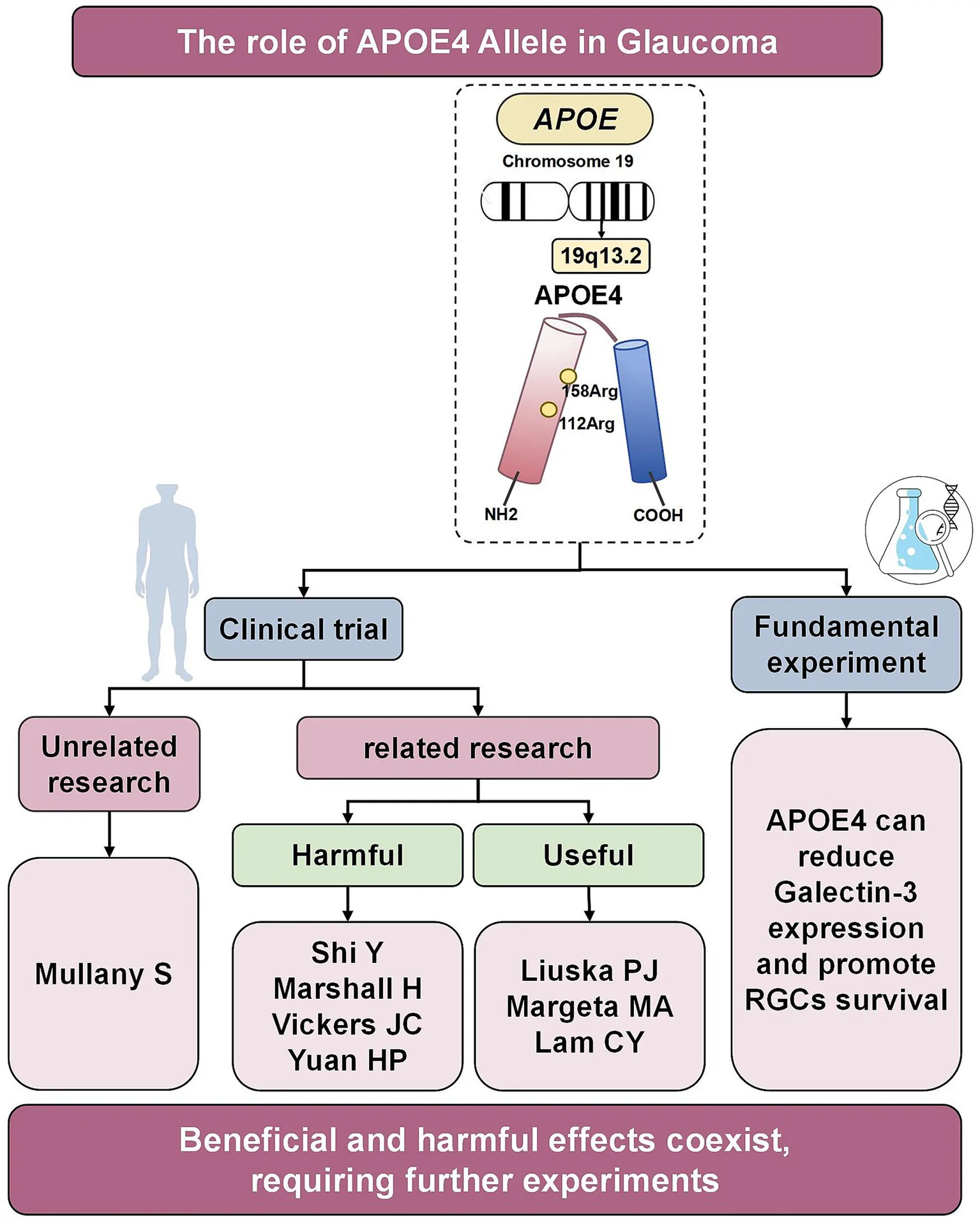
Schema 2: clinical and experimental evidence on the association between ApoE4 and glaucoma. The schema lists the studies discussed in Section 2.1 with the first author.

### 3.2 Role of the ApoE4 allele in AMD

AMD is a complex retinal neurodegenerative disease that disrupts central vision, representing the most common cause of permanent blindness in the elderly. Early AMD is associated with pathological deposits (drusen) between Bruch’s membrane (BM) and the retinal pigment epithelium (RPE). Late-stage AMD is usually divided into non-neovascular (“dry” AMD) and neovascular (“wet” AMD) form.

ApoE4 can significantly reduce the risk of AMD, whereas ApoE2 increases this risk-an effect opposite to that observed in AD (55, 128–133). ApoE4 can be involved in alleviating retinal inflammation and neovascularization in AMD by suppressing the expression of C-reactive protein (CRP), C-C motif chemokine ligand 2 (CCL2), and vascular endothelial growth factor (VEGF) (134–137). ApoE4 can also promote ATP-binding cassette transporter A1 (ABCA1)-mediated cholesterol transport, and maintain cholesterol homeostasis in RPE cells, thus restricting drusen formation (138). A clinical study conducted by Bakbak B et al. showed that compared to patients with other genotypes, exudative AMD patients carrying ApoE4 experienced significantly better visual acuity improvement after ranibizumab, a recombinant humanised monoclonal antibody fragment that neutralises VEGF-A and is a standard intravitreal treatment for neovascular (“wet”) AMD, treatment (139). Taken together, the AMD data are more consistent than those for glaucoma: ApoE4 is associated with a lower risk of AMD, whereas ApoE2 is associated with a higher risk, an effect that is the mirror image of the situation in AD.

ApoE2 can activate the MAPK signaling pathway (p-ERK, p-JNK, and p-p38), leading to the upregulation of downstream target genes (e.g., VEGF, PDGF-BB, and b-FGF growth factors) and proinflammatory cytokines (e.g., TNF-α, IL-1β, and IL-6) (140). It may further lead to retinal inflammation, thus the development and progression of subretinal neovascularization in AMD (140). Additionally, ApoE2 can reduce cholesterol transport in the retina, resulting in increased ceramide levels in the RPE. Elevated ceramide can cause autophagic defects, and complement-mediated mitochondrial damage, which then drives redox-sensitive cysteine-mediated phase separation of ApoE2, ultimately promoting drusen formation (138).

In addition, ApoE4 may restrict drusen formation, while ApoE2 exerts a promoting role, and the difference may be related to their chemical structures. Homozygous ApoE4 (Arg-112, and Arg-158) and homozygous ApoE2 (cysteine-112, and cysteine-158) differ from each other only in single amino acid substitutions at positions 112 and 158 (141). ApoE4 has Arg at both positions 112 and 158, preventing it from forming disulfide bonds or dimers with other peptide groups. Instead, it forms smaller lipid particles that can transport cholesterol across the BM more effectively, maintain cholesterol homeostasis in the RPE, thereby hindering drusen formation (142–145) (see Figure 3).

**Figure 3:**
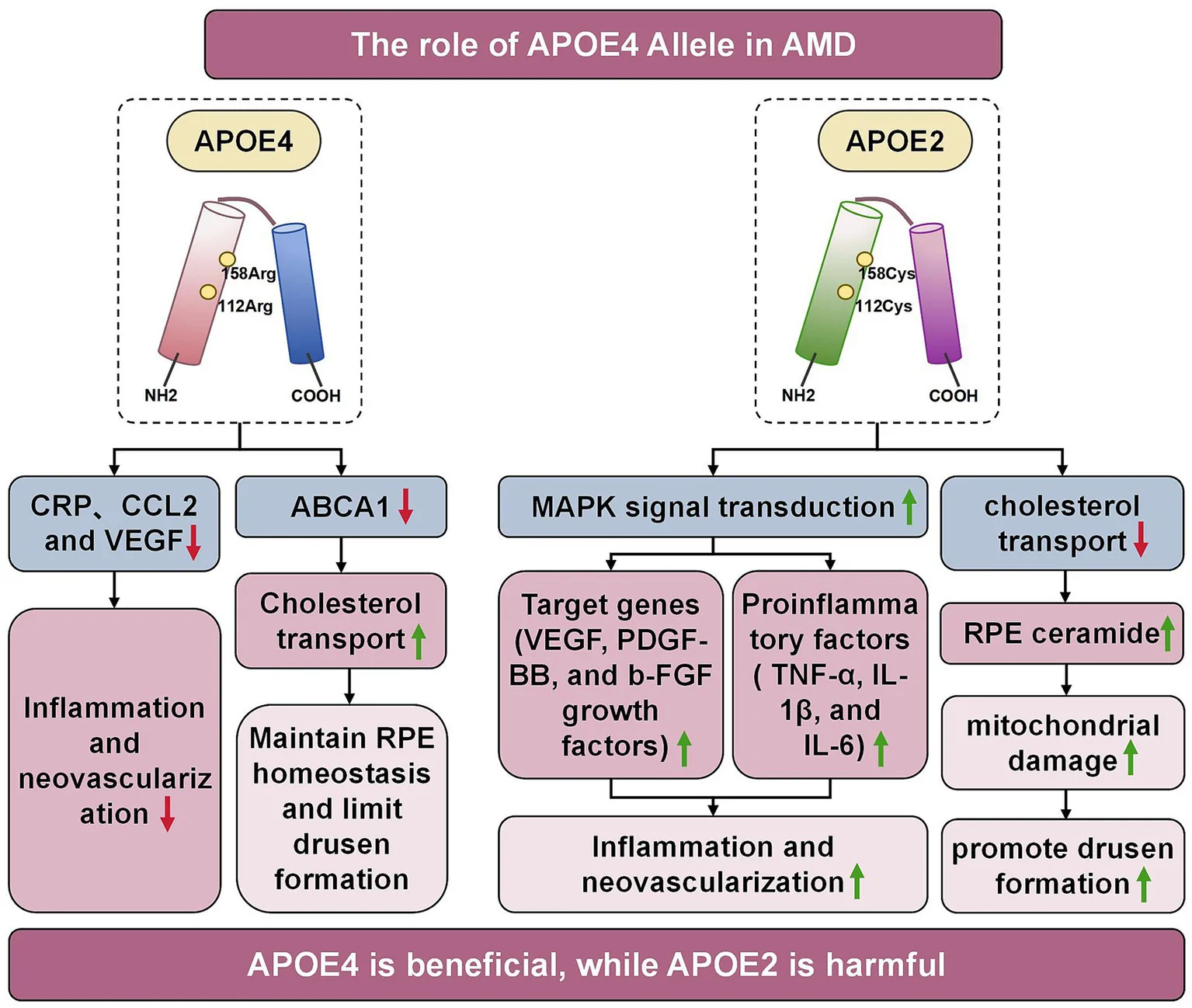
Opposing effects of ApoE4 and ApoE2 in AMD. ApoE4 suppresses CRP, CCL2 and VEGF expression, thereby limiting retinal inflammation and neovascularisation, and promotes ABCA1-mediated cholesterol transport, thereby maintaining cholesterol homeostasis in RPE cells and restricting drusen formation. ApoE2 activates the MAPK pathway (p-ERK, p-JNK, p-p38), upregulating VEGF, PDGF-BB and b-FGF and pro-inflammatory cytokines (TNF-α, IL-1β, IL-6), and reduces retinal cholesterol transport, leading to ceramide accumulation, autophagic defects and complement-mediated mitochondrial damage in the RPE, which together promote drusen formation and neovascularisation (referred to in Section 2.2).

### 3.3 The role of the ApoE4 allele in DR

DR is a major ocular complication of diabetes mellitus (DM), affecting approximately 30 to 40% of individuals with diabetes (146). Clinically, patients may usually experience retinal microvascular damage, including microaneurysms, hemorrhages, and hard exudates (146). Historically, research on early-stage DR has attached great attention to vascular abnormalities (146, 147). However, with advancements in retinal imaging modalities (e.g., optical coherence tomography angiography) and functional assessment techniques (e.g., electroretinography), retinal neural dysfunction is discovered to occur concurrently with vascular anomalies, and in some cases, even precede the development of vascular abnormalities (148–151). Collectively, a term of “diabetic retinal neurodegeneration”, falling within the spectrum of retinal neurodegenerative diseases, emerges to summarize these structural and functional changes-encompassing neural damage alongside vascular impairmen (148–151).

In the context of DM, the ApoE4 allele can increase the risk of DM, particularly in individuals with dyslipidemia (152). Boung Chul Lee and colleagues found in their cross-sectional study that DM patients who were ApoE4-positive would encounter an elevated risk of AD, whereas there was no significant association between ApoE4-negative DM and AD (153).

Additionally, current research has yielded conflicting results regarding the risk of DR associated with the ApoE4 allele. For example, a cross-sectional study Liew G et al. revealed no obvious association of the ApoE genotype with DR in White or African American patients with type 2 DM (T2DM) (154). A case–control study on the Czech population by Dlouha L and colleagues revealed a lower risk of developing DR in female T2DM patients carrying the ApoE4 genotype (155). Additionally, Tomomi Masuda et al. Indicated in their research that ApoE2 and ApoE3 could promote VEGF-induced cell proliferation and migration in DR to facilitate neovascularization, while ApoE4 did not exhibit such function and might reduce the risk of retinopathy in T2DM patients (156). However, a clinical study in Mexico by Arturo Santos and colleagues demonstrated a higher frequency and severity of macular edema and retinal hard exudates in DR patients carrying the ApoE4 genotype (157). As in glaucoma, therefore, the direction of the ApoE4 association in DR differs between cohorts, whereas the experimental data consistently indicate that ApoE2 and ApoE3, but not ApoE4, promote VEGF-driven retinal neovascularization (see Figure 4).

**Figure 4:**
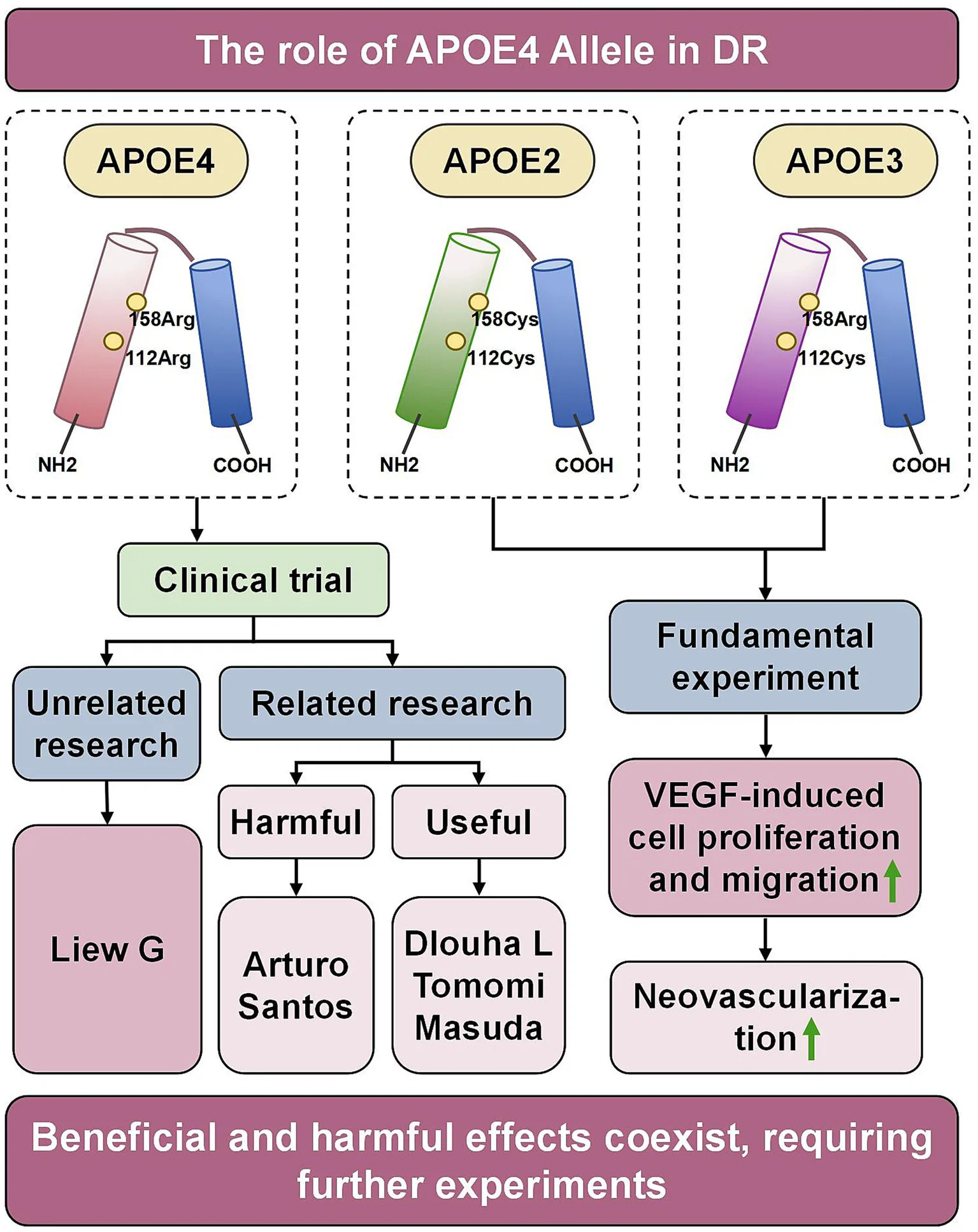
Association between ApoE genotype and diabetic retinopathy. Clinical studies of the ApoE genotype and DR are summarised with first author. The schema lists the studies discussed in Section 2.3.

## 4 ApoE4-targeted gene editing and gene therapy: current landscape, challenges and ophthalmic considerations

With the failure of drug research targeting AD in clinical trials—and given that the approved amyloid-targeting antibodies confer only modest clinical benefit—ApoE has emerged as an attractive therapeutic target. ApoE-directed gene editing and gene therapy are therefore being actively explored for AD; these are emerging and promising strategies. ApoE4 gene editing therapy has emerged as a mainstream direction for tackling AD in recent decades (158). Modifying ApoE from the ε4 allele to ε3 or ε2 via gene editing can effectively suppress ApoE4-associated disease risks (158). Treating neurons derived from ApoE4/4 human induced pluripotent stem cells (hiPSCs) with the ApoE4 structure corrector PH002 (36–38) has been discovered to ameliorate the adverse effects of ApoE4 on Tau phosphorylation, Aβ production, and GABAergic neuron degeneration in a dose-dependent manner (158, 159). Simultaneously, CRISPR/Cas9, the third-generation gene editing technology, has shown great potential in modifying the ApoE genotype (158–161). At the cellular level, cells derived from healthy individuals with the ApoE3/E4 genotype has been proven to be successful in converting to genotypes including ApoE2/E2, ApoE3/E3, ApoE4/E4, or ApoE knockout (158–161).

The available approaches fall into three mechanistically distinct classes. The first comprises small-molecule structure correctors, exemplified by PH002, which binds the pathological ApoE4 conformation and restores a more ApoE3-like fold (161). As noted above, treating neurons derived from ApoE4/4 human induced pluripotent stem cells (hiPSCs) with PH002 ameliorates the adverse effects of ApoE4 on Tau phosphorylation, Aβ production and GABAergic neuron degeneration in a dose-dependent manner (158). It should be emphasised that PH002 is a pharmacological tool compound used to establish proof of mechanism, not a therapeutic candidate.

The second class comprises RNA-based suppression. Antisense oligonucleotides, small interfering RNAs and vector-expressed artificial microRNAs can lower ApoE4 expression without altering the genome, are in principle reversible or titratable, and have already reduced ApoE expression and attenuated Tau and Aβ pathology in transgenic models (162–164). Their limitations are equally clear: the effect depends on continued dosing or sustained vector expression, the degree and durability of suppression in the human brain are unknown, and—critically for the present review—they reduce total ApoE rather than replacing it.

The third class comprises DNA-level interventions. Because the E3 and E4 alleles differ at single nucleotides, APOE is an attractive substrate for precision editing. Clustered regularly interspaced short palindromic repeat (CRISPR)/Cas9, the third-generation gene editing technology, has shown great potential in modifying the ApoE genotype: at the cellular level, cells derived from healthy individuals with the ApoE3/E4 genotype have been converted to ApoE2/E2, ApoE3/E3, ApoE4/E4 or ApoE knockout (165). Newer platforms improve on this in two respects. Base editors and prime editors install the desired sequence without relying on double-strand breaks, thereby reducing the risk of insertions, deletions and larger rearrangements; prime editing of the APOE4 allele to an APOE3-like sequence has recently been achieved in the brain of humanised APOE4 mice using brain-directed adeno-associated virus (AAV) vectors (166). Epigenetic editing, which silences or activates a allele without changing its sequence, offers a further, potentially reversible option (167). Taken together, the toolbox is now considerable; the unresolved problems lie less in the editors themselves than in delivering them safely, uniformly and to the right cells.

### 4.1 ApoE-targeted programmes in development

Several ApoE-directed programmes have now entered clinical or late preclinical development (Table 1). The most advanced is gene replacement rather than editing. LX1001 (Lexeo Therapeutics) is an AAV-based candidate designed to express the protective ApoE2 protein in the CNS of APOE4 homozygotes (168). It is being evaluated in an open-label, dose-escalation phase 1/2 trial and has received Fast Track designation from the US Food and Drug Administration for patients with early AD who are APOE4 homozygous (168). A complementary “reduce and replace” strategy is being pursued preclinically by Voyager Therapeutics: a bifunctional AAV that expresses an APOE-targeted artificial microRNA together with ApoE2 and is delivered intravenously using a blood–brain-barrier-penetrant capsid, achieving significant reduction of endogenous ApoE4 and concomitant ApoE2 expression in key AD-relevant brain regions of ApoE4 knock-in mice (169). At the academic end of the spectrum, AAV-delivered prime editing has been used to convert APOE4 to APOE3 in the brains of humanised APOE4 mice (169).

**Table 1:** Selected ApoE-targeted therapeutic programmes for AD.

Programme/sponsor	Modality and mechanism	Target population	Stage of development
LX1001/Lexeo Therapeutics (168)	AAV-mediated gene replacement; CNS expression of protective ApoE2	AD patients who are APOE4 homozygous	Phase 1/2, open-label dose escalation (LEAD); FDA Fast Track designation
Undisclosed bifunctional AAV / Voyager Therapeutics (170)	APOE-targeted artificial microRNA (knockdown of ApoE4) plus ApoE2 expression; BBB-penetrant capsid, intravenous delivery	AD (ApoE4 carriers)	Preclinical
AAV prime editing / academic (Weill Cornell Medicine) (166)	Prime editing of APOE4 to an APOE3-like sequence; dual AAV split-intein delivery to the brain	AD (ApoE4 carriers)	Preclinical
PH002 and related structure correctors (160)	Small molecule that corrects the pathological ApoE4 conformation	Not applicable (tool compound)	Preclinical; research tool, not a therapeutic candidate

### 4.2 Remaining challenges

Four groups of problems currently limit translation.

#### 4.2.1 Delivery

The brain and the retina are pharmacologically privileged compartments, and the ApoE pools within them are largely separate from the periphery; systemic editing of hepatic APOE would therefore not be expected to change CNS ApoE, whereas CNS-directed editing requires either direct administration into the parenchyma or the cerebrospinal fluid or a capsid capable of crossing the blood–brain barrier. Achieving widespread, cell-type-appropriate and dose-limited transduction in an adult human brain remains unsolved (171), and vector doses high enough to do so raise concerns about capsid-directed immunity and complement activation (172).

#### 4.2.2 Precision and durability

Off-target editing, bystander editing, mosaicism, and the long-term genomic stability of an irreversible edit in post-mitotic neurons all require rigorous assessment; the distribution of editing efficiency across brain regions in the prime-editing study cited above was uneven, with minor differences in the cerebellum (166).

#### 4.2.3 Biological uncertainty

ApoE is not simply a risk factor but a lipid transporter with essential roles in membrane repair, synaptic maintenance and barrier integrity, and complete loss of ApoE in humans is associated with type III hyperlipoproteinaemia; whether chronically lowering or replacing ApoE4 in the ageing brain is safe therefore remains an open question (173).

#### 4.2.4 Trial design

The field still lacks consensus on the optimal disease stage, the treatment window, and the biomarkers that would demonstrate target engagement and disease modification in APOE-stratified populations (174–176).

### 4.3 Implications for patients with coexisting retinal neurodegenerative disease

As shown in the table below regarding the role of ApoE4 in AD and retinal degenerative diseases (Table 2), ApoE4 is identified to be an absolute risk factor for AD. It has a clear protective effect in AMD, while there are currently conflicting results regarding its role in glaucoma and DR.

**Table 2:** The role of ApoE genotypes in AD and retinal degenerative diseases.

Disease	Genotype	Harmful	Useful
AD	ApoE4	√	-
	ApoE3	-	√
	ApoE2	-	√
Glaucoma	ApoE4	√	√
	ApoE3	-	-
	ApoE2	-	-
AMD	ApoE4	-	√
	ApoE3	-	-
	ApoE2	√	-
DR	ApoE4	√	√
	ApoE3	√	-
	ApoE2	√	-

Meanwhile, genetic editing of ApoE4 to ApoE3 may increase the risk of DR. Genetic editing of ApoE4 to ApoE2 may raise the risk of AMD. Complete deletion of the ApoE gene via genetic editing, known as ApoE knockout, may increase the risks of glaucoma, AMD, and DR. It may be explained that ApoE4 exerts varying degrees of protective effects against these three diseases. The practical implication is that not all ApoE-directed strategies carry the same ocular risk profile. Altogether, based on existing literature, it can be confirmed that we should be highly cautious in the consideration of ApoE4 gene editing therapy when AD occurs concurrently with retinal degenerative diseases.

Viewed through the lens of Table 1, the strategies now in development are not equivalent with respect to the eye. Complete deletion of APOE, or a strategy whose net effect is to reduce total ApoE in the retina, would remove whatever protection ApoE4 confers and might be expected to increase the risk of glaucoma, AMD and DR simultaneously. Conversion of ApoE4 to ApoE2, or ApoE2 overexpression, carries a different concern: ApoE2 promotes drusen formation and activates the MAPK-dependent, VEGF-driven pathways that underlie choroidal neovascularisation in AMD (138), and ApoE2 also promotes VEGF-induced proliferation and migration in DR (156); the retina may therefore be exposed to a pro-angiogenic and pro-inflammatory isoform if the transgene product reaches ocular tissue. Precision conversion of ApoE4 to ApoE3 preserves the total ApoE pool and avoids ApoE2-related angiogenic signalling, but it removes any ApoE4-specific protection and, on the evidence summarised in Section 2.3, may increase DR-related risk, since ApoE3—like ApoE2—promotes VEGF-induced neovascularisation whereas ApoE4 does not (156). On present evidence, therefore, isoform-preserving conversion to ApoE3 appears preferable to either knockout or ApoE2 overexpression for patients with co-existing retinal disease, although this inference is based largely on genetic association and preclinical data and requires direct experimental confirmation.

Route of administration matters as much as the molecular strategy. Experience from ocular gene therapy provides a cautionary signal: in a systematic review of 120 trials and 32 publications, intravitreal delivery of AAV was associated with substantially higher rates of anterior uveitis (43.06% versus 10.22%) and of intermediate or posterior uveitis (40.36% versus 6.18%) than subretinal delivery (177), and severe inflammatory events with vision loss have been reported after high-dose intravitreal AAV in eyes already compromised by diabetic macular oedema (178). Because AMD and DR are themselves characterised by a disrupted blood–retinal barrier and a primed inflammatory state, the diseased retina may be more, not less, susceptible to vector- or transgene-driven inflammation. Two practical conclusions follow. First, for AD patients with co-existing retinal disease, CNS-directed routes that minimise ocular exposure—intra-cerebrospinal fluid, intraparenchymal or intrathecal administration, or a systemically administered CNS-tropic capsid with demonstrated low ocular biodistribution—should be preferred over intravitreal or subretinal delivery, and ocular biodistribution should be an explicit preclinical requirement. Second, ophthalmic assessment should be built into these trials: baseline and periodic best-corrected visual acuity, fundus examination, optical coherence tomography and optical coherence tomography angiography, and—where the programmes target patients with diabetes—specific monitoring for macular oedema and hard exudates.

### 4.4 Future research directions

Three lines of investigation would most rapidly reduce the current uncertainty. First, mechanism: the protective effect of ApoE4 in the retina needs to be defined at the cellular level, and the Galectin-3/MGnD axis described in Section 2.1, together with isoform-specific effects on RPE cholesterol handling and VEGF signalling, offers a tractable starting point. Second, models and measures: humanised APOE mice, hiPSC-derived retinal organoids and RPE/choroid co-cultures carrying defined APOE genotypes should be used to test the retinal consequences of each editing strategy directly, rather than inferring them from genetic association studies; retinal imaging endpoints may additionally serve as accessible, non-invasive surrogate measures of neurodegeneration. Third, clinical translation: APOE-directed trials should stratify participants by both APOE genotype and retinal status, incorporate ophthalmic endpoints and long-term follow-up, and be linked to registries that capture ocular outcomes; inducible or otherwise regulatable expression systems would provide an additional margin of safety should ocular toxicity emerge. Until such data are available, the prudent position is that ApoE-directed interventions remain investigational, and that their use in AD patients with co-existing glaucoma, AMD or DR warrants explicit ophthalmological assessment and shared decision-making.

## 5 Problems and prospects

Currently, there have been extensive and in-depth research on the role of ApoE4 in the pathogenesis of AD. However, we currently know little about its role in retinal degenerative diseases such as glaucoma, AMD, and DR, and as outlined in Section 3, the programmes now entering the clinic have rarely considered ocular outcomes explicitly. Undoubtedly, existing studies provide sufficient and robust evidence to support that ApoE4 gene editing therapy should be selected cautiously and appropriately when AD is accompanied by retinal degenerative diseases. Moreover, it highlights the necessity for developing tailored treatment plans to address the overlapping pathophysiology of AD and retinal degenerative diseases, including genotype- and retina-guided choice of editing strategy, CNS-restricted delivery, and structured ophthalmic monitoring. Our team will continue to conduct more comprehensive research on unveiling the relationship between ApoE4 and AD as well as retinal degenerative diseases, so as to provide potential effective therapeutic strategies.
